# AQP5 regulates the proliferation and differentiation of epidermal stem cells in skin aging

**DOI:** 10.1590/1414-431X202010009

**Published:** 2020-09-18

**Authors:** Jing Zhou, Yabing Dong, Jianlan Liu, Jie Ren, Jinyan Wu, Ningwen Zhu

**Affiliations:** 1 Department of Dermatology, Huashan Hospital Fudan University, Shanghai, China; 2 Department of Oral Surgery, Shanghai Jiao Tong University School of Medicine, Ninth People's Hospital, Shanghai, China

**Keywords:** Skin aging, Epidermal stem cell, AQP5, Proliferation, Differentiation

## Abstract

The epidermis, the outermost layer of the skin, is the first barrier that comes into contact with the external environment. It plays an important role in resisting the invasion of harmful substances and microbial infections. The skin changes with age and external environmental factors. This study aimed to investigate epidermal stem cells during the process of aging. This study enrolled 9 volunteers with benign pigmented nevus for clinical dermatologic surgery. The phenotypes associated with skin aging changes such as skin wrinkles and elasticity of the unexposed/healthy parts near benign pigmented skin were measured, and epidermal stem cells from this region were isolated for transcriptome sequencing. The results showed that epidermal stem cells could be obtained by magnetic activated cell sorting (MACS) with high purity. Results of the transcriptome sequencing revealed that aquaporin (AQP)5 significantly decreased in the epidermal stem cells with age, and further functional experiments revealed that AQP5 could promote the proliferation and dedifferentiation of HaCaT, but did not influence cell apoptosis. In summary, AQP5 regulated the proliferation and differentiation of epidermal stem cells in skin aging, and it may play an important role in the balance of proliferation and differentiation. However, further studies are needed to determine the mechanism by which AQP5 regulates the proliferation and differentiation of epidermal skin cells in aging.

## Introduction

The skin is the outmost barrier and is the organ with the largest surface area of the body. It protects the body from physical and chemical damage, invasion of pathogenic microorganisms, and other environmental damage. It also plays an important role in maintaining body temperature and preventing water loss. However, with age, symptoms of aging gradually appear on the skin, such as wrinkles, thinning, and relaxation changes (1,2); furthermore, there are also changes in tissue structure (epidermal atrophy and epidermal cell layer reduction), and physiological function degradation and loss (3). Together with these symptoms, the skin's reactivity is weakened, reducing its defense against mechanical and chemical damage, microbial invasion, and regenerative healing ability, ultimately leading to a series of skin-related diseases (4-6).

The skin is composed of the epidermis, dermis, and subcutaneous tissue. The epidermis plays important roles in defending the body from environmental damage, such as UV exposure, which causes the skin to age via oxidative stress, and smoking. The epidermis contains the basal layer, spinous layer, granular layer, and stratum corneum. Epidermal stem cells (ESCs), which are located in the basal layer of the epidermis, have the ability of lifelong self-renewal, can maintain a constant number through division, while continuing to differentiate into various layers of skin tissue. Hence, the epidermis is in a state of continuous proliferation, differentiation, and apoptosis (2). ESCs are excessively depleted with aging, and no longer maintain epidermal tissue homeostasis and repair damaged tissues (7,8). For example, Wnt1 induces the hyperproliferation of hair follicle cells and the rapid exhaustion of stem cells (9), and p16^INK4a^ governs the processes of stem cell self-renewal by inhibiting the G1/S-phase transition of the cell cycle (10).

Aquaporins (AQPs) are a family of homologous water- and glycerol-transporting proteins expressed in many mammalian epithelial, endothelial, and other types of cells. AQPs have shown to play a role in transcellular permeability in many organisms (11,12). For example, AQPs play a role in secretory glands, such as salivary (13,14), lacrimal (15), and sweat (16) glands, and pancreatic (17,18) glands, and their role in secretory glands has been evaluated in AQP-null mice. Reduced perinatal survival in knockout mice and altered salivary function were found in AQP5-null mice (19), and null mice also exhibited a 60% reduction in saliva formation after pilocarpine stimulation (20). The number of active sweat glands was dramatically reduced after pilocarpine stimulation in AQP5 null mice (16). AQP5 seems to play an important role in secretion of some glands; however, no studies have reported the role of AQP5 in the epidermis.

These reports cited above indicate that the ESCs play a vital role in skin function and aging. To further understand the processes involved in skin aging, we studied changes in the ESCs during skin aging. In this present paper, we found that AQP5 may play an important role in maintaining the pluripotency of epidermal stem cells.

## Material and Methods

### Collection of clinical phenotypes and biopsy

Nine healthy volunteers (men=4; women=5; age=14-75 years [44±22]) who underwent dermatoplastic surgery for benign pigmented nevus between March and August 2019 at Huashan Hospital, Shanghai, were enrolled in this study. After arrival and check-in procedures, subjects were instructed to sit quietly to equilibrate to the 20±2°C room temperature and 50±5% humidity for 30 min. Instrumental measurements were conducted on each subject at a non-exposed site (abdomen, back, and thigh) surrounding the skin lesions. The skin wrinkles and elasticity were measured and analyzed by the Skin-Visioscan (Courage & Khazaka Electronic, Germany) and Cutometer^®^ MPA580 (Courage & Khazaka Electronic) individually. Samples of normal skin (0.5×0.5 cm^2^) surrounding the skin lesions were collected. All patients signed an informed consent to participate in this study.

### Primary epidermal stem cells isolation

The samples came from the non-exposed normal skin tissues near the region that had the lesion removed by dermatoplastic surgery for benign pigmented nevus. The samples were washed with sterile PBS 3 times and the tissues surrounding the epidermis were removed, especially the hypodermis. The remaining tissues were digested overnight at 4°C with DispaseII (Roche, Switzerland), and the entire epidermis, which was carefully peeled from the dermis and washed with sterile PBS, was digested with 0.25% Trypsin-EDTA (Thermo, USA) at 37°C for 5 min. After being sieved in 70- and 40-μm filters (Merck Millipore, USA), primary epidermal stem cells were isolated and purified by magnetic activated cell sorting (MACS: Miltenyi Biotec GmbH, Germany).

### Cell culture

Isolated primary epidermal stem cells were cultured with CNT medium (CELLnTEC, Switzerland) containing 10 µM of Y-27632 in 10-cm dishes pretreated with coating matrix (Gibco, USA), and incubated at 37°C in air containing 5% CO_2_. HaCaT cells (immortalized human keratinocyte cell line) were cultured in Dulbecco's modified Eagle's medium (DMEM) with 10% FBS (fetal bovine serum) and penicillin-streptomycin, and cells were incubated at 37°C with 5% CO_2_. AQP5 and the pCDH plasmid were transfected into the HaCaT cell line via lentivirus by Lipofectamine^®^ 2000 reagent (Invitrogen, USA).

### Immunofluorescence staining

Cells were seeded onto coverslips, then fixed with 4% paraformaldehyde for 30 min at room temperature, washed in phosphate-buffered saline (PBS), and then blocked in 2% (w/v) bovine serum albumin (BSA) in PBS for 30 min. The cells were stained overnight with primary antibodies at 4°C, followed by incubation with secondary antibody for 1 h at room temperature, then stained with 4′,6-diamidino-2-phenylindole (DAPI) for 30 min. The primary antibodies included anti-cytokeratin 14 antibody (Abcam, UK), CD49f antibody (Abcam), anti-filaggrin antibody (Abcam), anti-P63 antibody (Abcam), and anti-Ki67 antibody (Abcam). The secondary antibodies included anti-mouse IgG (CST, USA) and anti-rabbit IgG (CST). For the proliferation assay, immunofluorescence staining was carried out according to the protocol of Cell-Light EdU Apollo567 *in vitro* kit (RIBOBIO, China), and imaging and analysis were performed using Harmony^®^ 4.8 (PerkinElmer, USA).

### RNA sequencing

Isolated primary epidermal stem cells were cultured with CNT medium and used for RNA sequencing. RNA libraries were processed according to the manufacturer's protocol, using Illumina NEB Next^®^ Ultra™ RNA Library Prep Kit (Illumina, USA). RNA purification, fragmentation, primer hybridization, and sequencing reactions were performed according to the manufacturer's protocol. High-throughput sequencing was performed on an Illumina HiSeq 2000 platform. FPKM (fragments per kilobase of transcript per million mapped reads) values for genes were calculated via Cufflinks v1.2.1 (http://cole-trapnell-lab.github.io/cufflinks/). Hierarchical cluster analysis, principal components analysis (PCA), and volcano plot analysis were performed using R software (www.r-project.org).

### RT-qPCR

Total RNA was isolated from samples using Trizol reagent (Invitrogen, USA), and the resultant cDNA was reversely transcribed using PrimeScript TM RT Master Mix (TaKaRa, Japan). PCR was performed using ChamQ Universal SYBR qPCR Master Mix (Vazyme, China) on an ABI7900 Real-Time PCR system (Applied Biosystems, USA). *GAPDH* and *β-actin* were used as the internal references, and mRNA expression was calculated using the 2^-ΔΔCT^ method. Table 1 lists the primers used for the RT-qPCR.

**Table 1:** Primers used in RT-qPCR.

	Forward Primer (5′-3′)	Reverse Primer (5′-3′)
*AQP5*	TCTCTCTGGGACAGACCTCA	TGGGATCCCTTCAGCTCTTG
*ARC*	AGTCTTTGCTGCTGTGCT	CAGCCGACTCCTCTCTGTAG
*AIRE*	AGTGCTGAGAAGGACACCTC	CTGTTTACAGCCGAGCACTG
*KLF2*	ACCTACACCAAGAGTTCGCA	CACAGATGGCACTGGAATGG
*EPPK1*	ATCTATGAGGCCCGATGCAA	CACATCAGGCCCAATGACAG
*ID4*	GTGAGTAGTACCGGGAGTGG	TCCTAGTCACTCCCTTCGGA
*KRT1*	AGAGTGGACCAACTGAAGAGT	ATTCTCTGCATTTGTCCGCTT
*KRT5*	AGGAGTTGGACCAGTCAACAT	TGGAGTAGTAGCTTCCACTGC
*KRT10*	GACGTAATGTACAAGCTCTGGA	TGGGCCTGAATCTGTGAGAG
*KRT14*	TGAGCCGCATTCTGAACGAG	GATGACTGCGATCCAGAGGA
*LOR*	CATTGCCAGCATCTTCTCTCCT	AGAGGTCTTCACGCAGTCCA
*β-actin*	ACTCTTCCAGCCTTCCTTCC	CAATGCCAGGGTACATGGTG
*GAPDH*	GAAGGTGAAGGTCGGAGTC	GAAGATGGTGATGGGATTTC

### Statistical analysis

Statistical analyses were performed and statistical graphs were obtained using GraphPad Prism 6.01 software (USA). Student’s *t*-test was used to compare the two groups. P<0.05 was considered significant.

## Results

### Skin aging phenotype in young and elderly groups

**Figure 1:**
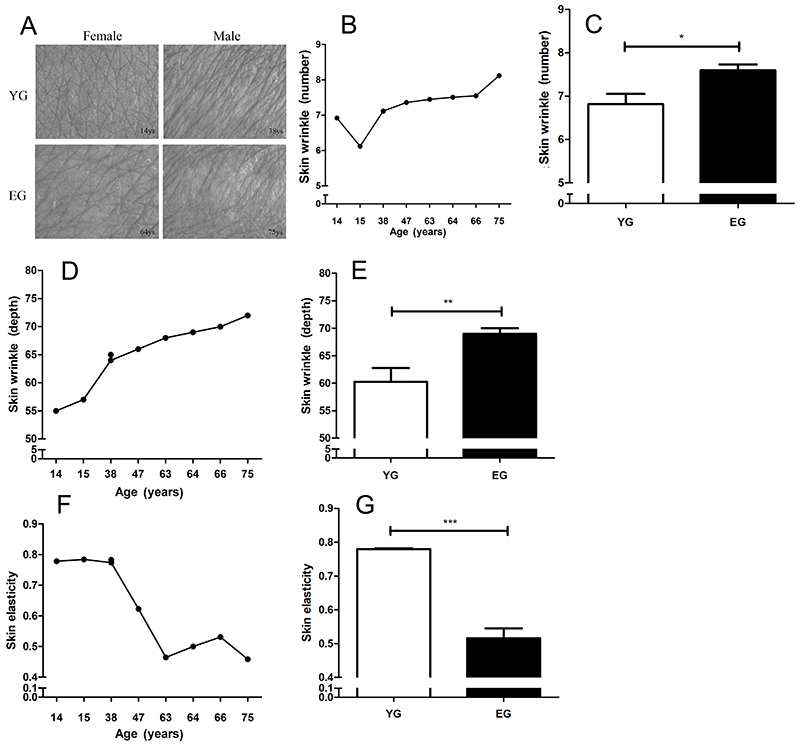
Skin wrinkles and elasticity in the young and elderly groups (**A**). Images of the abdomen from a male and a female, of the young group (YG, 0-40 years) and elderly group (EG, 41-80 years). Number (**B**) and depth (**D**) of wrinkles on the skin according to age of volunteers, and according to age groups (**C** and **E**). Panel B: The data for two volunteers overlapped (7.12 and 7.11). Skin elasticity value for every volunteer (**F**) and comparison of the skin elasticity between age groups (**G**). *P<0.05, **P<0.01, ***P<0.001 (*t*-test). Data are reported as mean±SD.

To investigate the effect of age on skin phenotypes and epidermal stem cells, we divided the sample into two group: young group (YG, ≤40 years) and elderly group (EG, ≥41 years). Images of normal skin tissue are shown in Figure 1A. The number (Figure 1B) and depth (Figure 1D) of wrinkles increased with age, and there was a significant difference in the number of wrinkles (Figure 1C) and depth (Figure 1E) between the YG and EG, indicating that skin wrinkles increase, deepen, and become rough with age. The results also showed that skin elasticity decreased with age (Figure 1F and G).

### Isolation of epidermal stem cells

**Figure 2:**
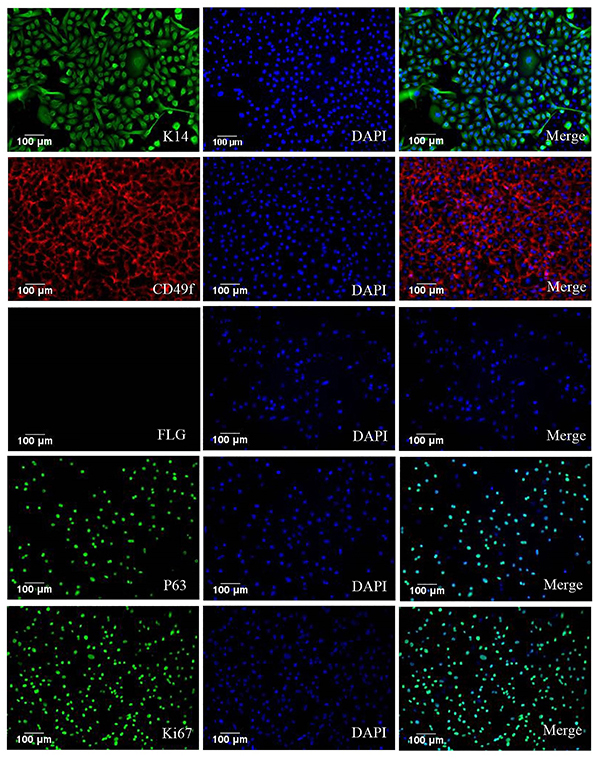
Immunofluorescence staining of K14, CD49f, filaggrin, P63, and Ki67 in epidermal stem cells sorted by magnetic activated cell sorting. Blue represents Hoechst-stained cells. Magnification bar: 100 μm.

To further study changes in the epidermis during skin ageing, we isolated epidermal stem cells from normal tissue using MACS. The cell purity was 84.50±1.95% and cell activity was 81.06±3.56% (data not shown). To confirm that the isolated cells were ESCs, ESC markers were used for immunofluorescence staining, and the isolated cells were positive for the ESC markers K14, P63, and CD49f. Moreover, Ki67 staining indicated that the isolated cells had the potential for proliferation and were negative for the differentiation marker filaggrin (Figure 2).

### Differential expression of genes in two age groups

**Figure 3:**
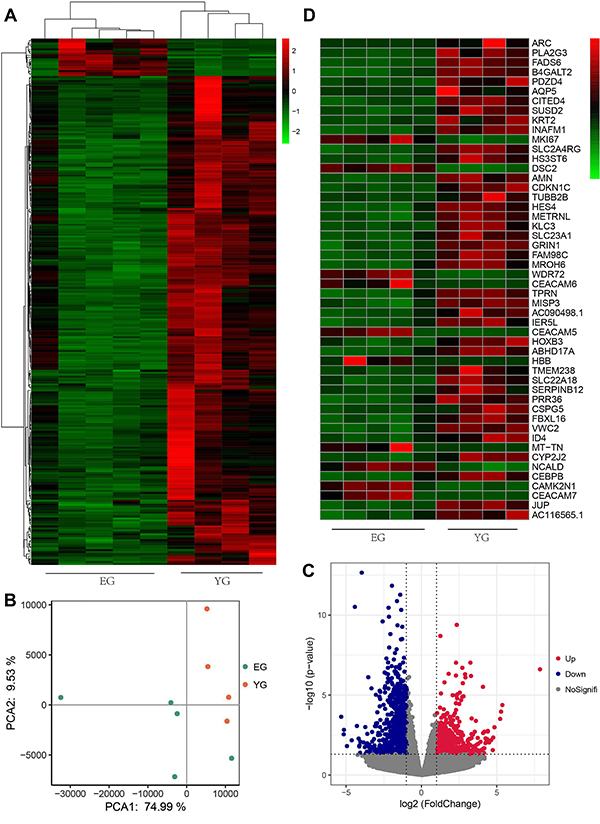
Differentially expressed genes (DEGs) between the young group (YG) and elderly group (EG) groups identified by RNA-seq analysis. **A**, Heatmap and hierarchical clustering analysis. The colors red to green represented the gene expression level. **B**, Results of the principal component analysis: orange dots represent samples of the YG group (n=4) and the cyan dots represent the EG group (n=5). **C**, A volcano plot of DEGs. Up- and downregulated DEGs with P <0.05 and a fold change >2 are represented in red and blue, respectively. **D**, The heatmap shows the top 50 DEGs with significant differential expression from panel **C**.

To investigate changes in the YG and EG groups, RNA sequencing was carried out. The heatmap illustrated differentially expressed genes (DEGs) (Figure 3A). PCA showed two significant clusters in the two groups (Figure 3B), indicating that the similarity of the samples belonging to the same group was higher than that belonging to a different group. A total of 443 DEGs exhibited a fold change >2 and a P value <0.05 (Figure 3C), of which 89 were upregulated and 354 were downregulated. The top 50 DEGs are illustrated in Figure 3D.

### AQP5 regulated the proliferation and differentiation in HaCaT cells

**Figure 4:**
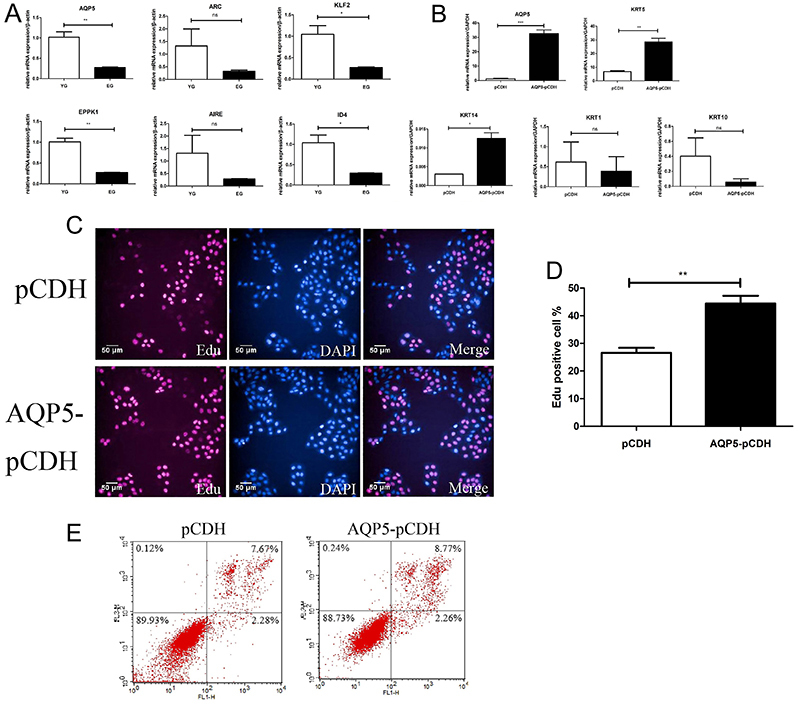
AQP5 regulated the proliferation and differentiation in HaCaT cells. **A**, Validation of differentially expressed genes based on the RNAseq results. **B**, The mRNA expression levels of *AQP5*, *KRT1*, *KRT5*, *KRT10*, and *KRT14* in HaCaT cells were assessed by real-time qRT-PCR after cells were infected with an *AQP5* overexpression vector. **C** and **D**, Immunofluorescence staining to quantify the proliferation in the *AQP5* overexpression cell line and compare it to the control (magnification bar: 50 μm). Red represents positive cells, blue represents DAPI. **E**, Apoptosis in the *AQP5* overexpression cell line compared to the control. Data are reported as mean±SD. *P<0.05, **P<0.01, ***P<0.001 (*t*-test). ns: not significant.

To further investigate the genes that changed between the two groups, we validated DEGs from the RNAseq results (Figure 4A). Further confirmation showed that AQP5, KLF2, EPPK1, and ID4 were markedly decreased in the EG group, but the *ARC* and *AIRE* genes did not significantly differ. Therefore, as the *AQP5* gene was the most significantly expressed gene in this study, we investigated its function in HaCaT cells. We first constructed a plasmid overexpressing *AQP5* with stable expression in HaCaT cells, and measured the differentiation genes in the epidermis by RT-qPCR (Figure 4B). The results showed that *KRT5* and *KRT14*, the basal layer expression genes, were upregulated, and *KRT1* and *KRT10*, the spinous layer expression genes, did not change. Furthermore, the granular expression gene *loricrin* was underestimated. Immunofluorescence staining indicated that the *AQP5* gene can promote cell proliferation when it is overexpressed (Figure 4C and D), and flow cytometric results revealed that AQP5 cannot influence cell apoptosis (Figure 4E). The results indicated that *AQP5* could control the proliferation and differentiation in HaCaT cells, but it had no effect on apoptosis.

## Discussion

In the present study, we observed the following: 1) ESCs were successfully isolated and RNAseq sequencing was performed to identify the DEGs between two different age groups; and 2) *AQP5* could regulate the proliferation and differentiation of HaCaT cells and may play an important role in maintaining the potential of ESCs.

Skin aging is divided into extrinsic and intrinsic aging (4). Extrinsic aging occurs due to various environmental factors such as UV exposure and smoking. Photoaging is caused by UV light and can lead to deep wrinkles, loss of skin elasticity, and skin pigmentation (3). Histological changes, thickening of the epidermis, and uneven distribution of melanocytes have also been observed in photodamaged skin (3). The primary mechanism of damage is UVA and can cause the generation of reactive oxygen species (ROS), which damage the DNA, lipid, and protein (21). However, intrinsic aging mainly manifests as non-exposed skin features such as wrinkles, skin sagging, and dryness (6,22), these features may be caused by hormonal changes, estrogens, and androgens (23-25). ROS are another cause of intrinsic aging (26), similar to extrinsic aging. ROS are continuously produced as byproducts in the electron transport chain of mitochondria during aerobic metabolism, and the abundant generation of superoxide anions may harm cellular function, leading to cellular senescence (8). Therefore, we measured wrinkles and elasticity in the skin at a non-exposed site (abdomen, back, and thigh) of volunteers, as these results could reflect the intrinsic aging state of the skin epidermis to some extent. The phenotype results of intrinsic aging revealed an increase in the number of wrinkles and decrease in skin elasticity with age, reflecting aging of the skin. Based on the RNAseq heatmap (Figure 3) results, the cluster could be divided into two groups using 40 years as the threshold; therefore, we divided the sample into two groups: a young group (1–40 years) and an elderly group (41–80 years). The number of skin wrinkles and depth showed a marked increase and the skin elasticity notably decreased in the EG (Figure 1B–G), showing intrinsic aging of the skin.

Isolated cells from the non-exposed normal tissues were stained using immunofluorescence staining for the markers K14, filaggrin, Ki67, CD49f, and P63. The isolated cells were positive for K14 and CD49f (basal layer cell markers (27)) and negative for filaggrin (granular layer cell marker). To further confirm that the isolated cells were ESCs, cells were tested by immunofluorescence staining of the stem cell marker P63 (28) and were found to be positive. Furthermore, immunofluorescence staining of Ki67 indicated that the isolated cells have high proliferation potential (Figure 2). In conclusion, we successfully isolated ESCs from the epidermis, which could be studied further.

Previous studies have reported AQP1, AQP3, AQP9, and AQP10 expression in the epidermis (29-31). AQP3 is located in the basal and spinosum layers (29), and deletion of the *AQP3* gene in mice resulted in dry skin (32). Furthermore, AQP3 can regulate keratinocyte migration, proliferation, and differentiation, which is associated with decreased metabolism. Previous reports have revealed that AQP5 increased keratinocyte chemoattractant expression, which may be mediated by increased ERK and NF-κB activation in NIH-3T3 cells (33). However, no study has reported the role of AQP5 in keratinocytes. In the present study, we discovered that AQP5 can regulate keratinocyte proliferation and differentiation. AQP5 overexpression in HaCaT cells could induce proliferation and dedifferentiation but not influence the apoptosis of HaCaT cells, suggesting that AQP5 mediated the balance between epidermal keratinocyte proliferation and differentiation and may play an important role in maintaining the potential of keratinocytes. A previous report demonstrated that skin wound healing was impaired in AQP3-deficient mice (34), and further investigations indicated that AQP3 deficiency in keratinocytes was associated with reduced levels of glycerol and ATP in epidermal cells, which resulted in keratinocyte proliferation defects that could be largely abolished by glycerol replacement (35). However, the mechanism by which AQP5 regulates the proliferation and differentiation of keratinocytes is not very clear and requires further research.

In conclusion, AQP5 promoted the proliferation of HaCaT cells while inhibiting their differentiation, indicating that AQP5 can maintain the potential of ESCs in skin aging. Based on these results, AQP5 could be a potential target for cosmetics and to prevent skin aging.
